# Persistent Human Metapneumovirus Infection in Immunocompromised Child

**DOI:** 10.3201/eid1405.071459

**Published:** 2008-05

**Authors:** Yacine Abed, Guy Boivin

**Affiliations:** * Centre Hospitalier Universitaire de Québec, Quebec City, Quebec, Canada; † Infectious Disease Research Centre, Quebec City, Quebec, Canada

**Keywords:** Human metapneumovirus, hMPV, persistence, G gene, letter

**To the Editor:** Respiratory viral infections can be associated with a wide range of clinical manifestations from self-limiting upper respiratory tract diseases to pneumonia (*1*). However, in general, respiratory viral infections are more likely to progress to more severe diseases in immmunocompromised patients. Human metapneumovirus (hMPV) has been reported in most parts of the world as a cause of acute respiratory tract infections in persons of all age groups (*2*). Fatal hMPV infections have been reported in immunocompromised patients, including a 17-month-old girl who had acute lymphoblastic leukemia (*3*) and a 33-year-old woman who had received a hematopoietic stem cell transplant (HSCT) (*4*). In adult HSCT recipients, fatal pneumonia (*5*) and persistent hMPV infection without respiratory symptoms have been described (*6*). In addition, adult lung transplant recipients have been able to clear hMPV infection despite high levels of immunosuppression (*7*). We report a case of persistent hMPV infection in a child with severe combined immunodeficiency disorder (SCID) who shed hMPV during an 11-month period.

The child, a girl who was born in January 2002, received an allogeneic haploidentical stem cell transplant from her father in May 2002 after her diagnosis of SCID. Infection with influenza A virus (H3N2) was diagnosed on April 2005 and progressed to a chronic pneumonitis of the lingula. She received successive courses of anti-influenza agents (amantadine, oseltamivir, and zanamivir) for 1 year during which time several positive influenza cultures were obtained (*8*). Four years after the transplant, she was still lymphopenic (800 × 10^9^/L, mostly T cells) and had chronic graft-versus-host disease, which had been treated with steroids (prednisone 2.5 mg twice a day for many months). She also had a mild chronic cough but did not need supplemental oxygen while she was receiving nebulized zanamivir (10–20 mg twice a day). Her 2 nasopharyngeal aspirate (NPA) specimens from June and July 2006 were negative for influenza virus. However, positive cultures for hMPV were obtained from NPA and bronchoalveolar lavage specimens collected on July 2006. After receiving this result, we performed retrospective and prospective molecular detection studies for hMPV for this patient. HMPV was detected by reverse transcription–PCR for the F and G genes (*9*) in 6 and 7 NPA samples, respectively, collected during an 11-month period from November 4, 2005, through October 4, 2006. These samples were obtained for surveillance of influenza infection in this child with persistent cough.

Amplified hMPV G sequences were aligned by using the ClustalW program (www.molecularevolution.org/cdc/software/clustalw). A phylogenetic tree was constructed with MEGA 3.1 software (www.megasoftware.net) by using the neighbor-joining algorithm with Kimura-2 parameters. Sequence analysis of the hMPV G gene showed that all strains belonged to the B2 genotype, which clustered with hMPV Can98–75 and NL1/94 reference strains (Figure, **panel A**). Amplified hMPV G gene sequences of the 6 samples collected in 2006 were identical, but they had 96.7% and 92.8% nucleotide and amino acid identities, respectively, with the initial strain from November 2005, which clearly indicates 2 viral strains (Figure, **panel B**). Similar results were obtained with the F gene (data not shown). Inoculation of the respiratory samples on a panel of 10 cell lines as previously described (*10*) showed that only 2 of 7 NPA samples were positive for hMPV by culture; 2 of the 5 remaining samples were positive for influenza A, which may have masked the cytopathic effects of hMPV on rhesus monkey kidney (LLC-MK2) cells.

**Figure:**
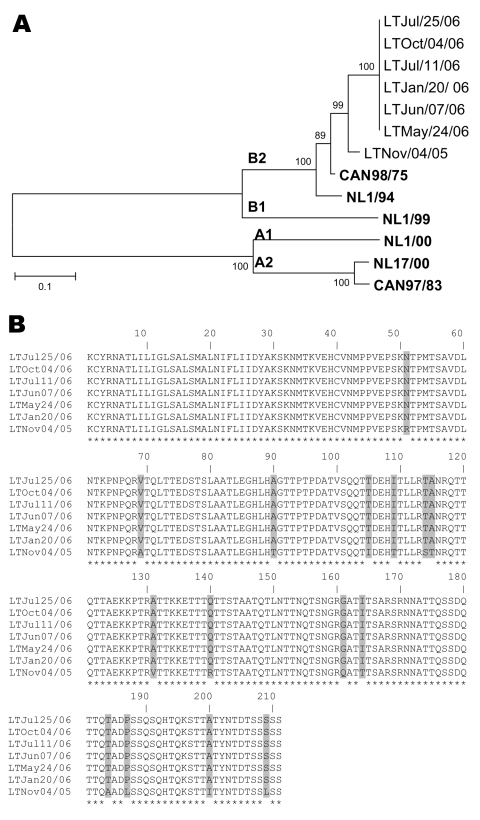
A) Phylogenetic analysis of human metapneumovirus (hMPV) strains isolated during an 11-month period based on nucleotide sequences of the G gene. Multiple nucleotide sequence alignments were performed by using the ClustalW program (www.molecularevolution.org/cdc/software/clustalw); a phylogenetic tree was constructed with MEGA 3.1 software (www.megasoftware.net) by using the neighbor-joining algorithm with Kimura-2 parameters. The analysis included the following hMPV reference strains: Can98/75 (GenBank accession no. AY485245), NL1/94 (AY304362), NL1/99 (AY304361), NL1/00 (AF371337), NL17/00 (AY304360), and Can97/83 (AY485253). Scale bar indicates 1 substitution for every 10 nucleic acid residues. **Boldface** indicates reference isolates. B) Comparison of the partial amino acid sequences (residues 26–236) of the G protein of hMPV isolates recovered during an 11-month period from an immunocompromised child. Asterisks denote identical residues; shaded boxes highlight different amino acids between the hMPV variant of November 4, 2005, and the subsequent variants from January 20, 2006, to October 4, 2006.

Persistent hMPV infection in asymptomatic adult HSCT recipients has been described (*6*). In that study, hMPV was isolated from 2 patients in 2 consecutive samples collected 12–56 days apart. However, virus evolution was not adequately investigated because it was based on sequence analysis of a 150-bp fragment from the highly conserved nucleoprotein gene (*6*). Unlike in previous reports (*6*,*7*), characterization of hMPV strains in our study was performed by sequence analysis of a 633-bp fragment from the most variable hMPV G gene. Our findings showed 2 distinct hMPV variants of the same genotype (B2). These variants might represent a viral drift after immune pressure but most likely was the result of 2 different infections in the immunocompromised child. The latter hypothesis is suggested by the considerable amino acid variability (15 aa differences in the 211-aa region of the G protein) between strains collected on November, 4, 2005, and January, 20, 2006, compared with identical sequences for the strains recovered over the next 10 months. Debiaggi et al. (*6*) previously suggested that persistent hMPV infection in HSCT patients was attributable to their inability to clear the virus because of impaired immune response. By contrast, adult lung transplant recipients were found to be able to achieve hMPV clearance despite their severe immunosuppression status (*7*). Because both fatal and mild or asymptomatic hMPV infections have been reported in immunocompromised hosts, additional studies are needed to determine whether such differing outcomes are due to viral, host, or environmental factors. In conclusion, this case of persistent hMPV infection associated with relatively mild respiratory symptoms in an immunocompromised child suggests that the host’s immune response may play a key role in disease pathogenesis.
